# Optical Flow Structure Effects in Children’s Postural Control

**DOI:** 10.1371/journal.pone.0158416

**Published:** 2016-06-28

**Authors:** Daniela Godoi, José A. Barela

**Affiliations:** 1 Department of Physical Education, Center of Biological Sciences and Health, Federal University of São Carlos (UFSCar), São Carlos, São Paulo, Brazil; 2 Institute of Physical Activity and Sport Sciences, Cruzeiro do Sul University (UNICSUL), São Paulo, São Paulo, Brazil; 3 Department of Physical Education, Institute of Biosciences, São Paulo State University (UNESP), Rio Claro, São Paulo, Brazil; Ludwig-Maximilian University, GERMANY

## Abstract

The aim of this study was to investigate the effect of distance and optic flow structure on visual information and body sway coupling in children and young adults. Thirty children (from 4 to 12 years of age) and 10 young adults stood upright inside of a moving room oscillating at 0.2 Hz, at 0.25 and 1.5 m from the front wall, and under three optical flow conditions (global, central, and peripheral). Effect of distance and optic flow structure on the coupling of visual information and body sway is age-dependent, with 4-year-olds being more affected at 0.25 m distance than older children and adults are. No such difference was observed at 1.5 m from the front wall. Moreover, 4-year-olds’ sway was larger and displayed higher variability. These results suggest that despite being able to accommodate change resulting from varying optic flow conditions, young children have difficulty in dodging stronger visual stimuli. Lastly, difference in sway performance may be due to immature inter-modality sensory reweighting.

## Introduction

Studies have showed developmental changes in children’s postural control throughout their first decade of life (e.g., [1–6]). Despite long-standing explanations attributing these changes to the manner in which sensory information is used to control posture (e.g. [7]), only recently has the relationship between sensory information and body dynamics control been examined in depth (e.g., [8–12]). In general, these studies indicate that although infants and children up to 8 years of age use sensory information to control posture, they do so in different ways as compared to older children and adults. Therefore, issues underpinning sensory and motor coupling in children are yet in need of clarification.

One of the strategies employed to investigate the use of sensory information is to manipulate available cues to one sensory channel—with cues to other channels remaining unchanged—and observe motor responses to sensory manipulation [13]. Lee and colleagues pioneered the use of this strategy with infants by manipulating visual cues in a moving room[14], and were followed by many others (e.g., [9, 12, 15]). Schmuckler [11] also employed the moving room strategy with 3- to 6-year-old children. In this study, frequency/speedy of visual stimulus was changed not only from a trial to another (Experiment 1) but also within a trial (Experiment 2). Results showed that besides swaying continuously to visual stimulus manipulation, children tempered postural responses after initial exposure to visual stimulus manipulation, indicating that even young children could adapt to visual stimulus manipulation changes.

Differently of these previous findings, Godoi and Barela [16] observed that 10-year-old and younger children standing inside of a moving room were not able to accommodate changes in structure of visual cues as a result of standing at different distances from the front wall. It was suggested that children before the age of 10 years could not reweight sensory cues in the same way as older children and adults did in order to offset changes in structure of available visual stimuli. Since the effect of visual cues diminished as young children stood father away from the frontal wall of the moving room, Godoi and Barela suggested that young children had more difficulty in detecting changes in retinal slip [16] and to adapt to these changes in order to produce correspondent body sway as older (children over 10-year-old) and adults do.

Several studies have examined the effect of different optic flow structures on postural control in infants [9, 17], children up to 5 years of age [9, 18], and adults [19, 20]. These studies indicated that infants as young as 7 months of age are influenced by global flow, but are only affected by central and peripheral flows several months later [9, 17, 18]. In addition, these results suggested that 5-year-old children and younger, like adults [19, 20], use visual cues from different flow structures to control their posture, but are more strongly affected by global and peripheral flows than by central flow.

Despite the aforementioned studies showing that infants and young children are able to respond to moving room manipulation, present understanding of children’s sensitivity to global motion is controversial, primarily because of the different speeds with which visual stimuli are presented [21, 22]. Since the moving room speed employed in Godoi and Barela’s study [16] was relatively low, it is possible that, in this case, young children could not detect its movement at a distance due to diminished retinal slip. Therefore, it seems reasonable to suggest that differences in sensitivity to changes in optic flow structure explain differences observed between children and young adults. Such a suggestion is in accordance to results from studies that investigated the use of central and peripheral vision cues in children [23, 24]. However, to our knowledge, no study has ever investigated optical flow sensitivity in children older than 5 years of age. For that reason, the aim of this study was to investigate the influence of distance and optic flow structure on visual information and body sway coupling in children and young adults. Specifically, this study sought to evaluate 4-, 8-, and 12-year-old children’s and young adults’ visual information and body sway coupling at two distances from the front wall, under central, peripheral, and global flow conditions.

## Materials and Methods

### Participants

Forty subjects, divided equally into four age groups: 4- (M = 4.28 and SD = 0.30), 8- (M = 8.59 and SD = 0.24), 12- (M = 12.27 and SD = 0.16), and young adults (M = 22.72 and SD = 1.95), participated in this study. Children were recruited at local schools, daycare centers, and through personal contact. The young adult cohort consisted of undergraduate and graduate students. Both young adults and children’s parents provided written informed consent prior to participation, according to procedures approved by the Institutional Review Board of Bioscience Institute, São Paulo State University (UNESP). None of the participants had any known neuromuscular problem that could hinder maintenance of upright position and all of them had normal or corrected-normal vision.

### Procedures

**Fig 1:**
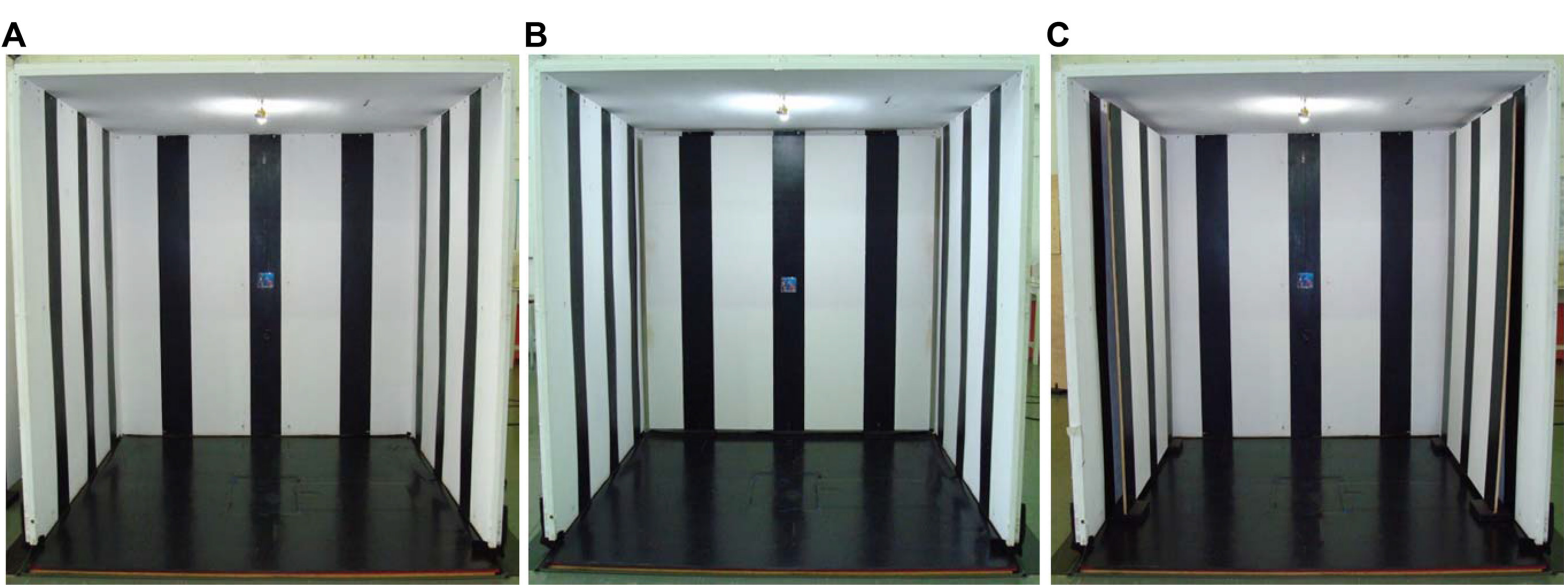
Pictures of the moving room. Pictures of the moving room showing the room in the global (A), peripheral (B), and central (C) optical flow conditions.

Participants were asked to maintain upright position inside of a moving room (Fig 1) and look at an infantile picture (0.1 x 0.1 m) attached to the front wall at eye level. The moving room—consisting of three walls and a ceiling (2.1 m long x 2.1 m wide x 2.1 m tall)—was mounted on wheels so that it could be moved back and forth by a servomotor mechanism. The walls and ceiling of the moving room were covered with a pattern of black and white vertical stripes, 22 cm and 42 cm wide, respectively. A 20-watt light bulb was used to maintain constant lighting from the ceiling throughout data collection. The servomotor mechanism consisted of a controller (Compumotor, Model APEX 6151), a controlled stepper motor (Compumotor, Model N0992GR0NMSN), and an electrical cylinder (Compumotor, Model EC3-X3xxN-10004a-Ms1-MT1M), which connected the servomotor to the moving room structure. Specialized software (Compumotor, Motion Architect for Windows) controlled the servomotor mechanism, moving the room away from and toward the participant (anterior-posterior direction). The moving room uninterruptedly oscillated backward and forward at 0.2 Hz frequency, 0.5 cm amplitude, and 0.6 cm/s peak velocity.

One OPTOTRAK (Northern Digital Inc.) IRED was affixed to the participants’ back (at approximately the level of the eighth thoracic vertebra) and another was attached to the front wall of the moving room. These markers provided information about the participants’ trunk sway and the moving room displacement, respectively, in anterior-posterior, medial-lateral, and vertical directions, collected at 100 Hz sampling rate.

All participants underwent three experimental visual stimulus conditions: global, peripheral, and central optical flow. Under the global optical flow condition, optical flow from the entire room (front and lateral walls and ceiling) was available to participants (Fig 1A). Under the peripheral optical flow condition, only optical flow from the lateral walls and ceiling was available. This was possible because, under this condition, a stationary wooden board, covered with the same pattern of black and white vertical stripes, was placed at 0.12 m away from the front wall of the moving room, preventing participants from receiving visual stimulus due to front wall oscillation (Fig 1B). Under the central optic flow condition, two stationary wooden boards, covered with the same black and white pattern, were placed 0.12 m away from each lateral walls (right and left sides) of the moving room, preventing participants from receiving visual cues due to oscillation of lateral walls (Fig 1C). For instance, optical flow available to the participant was either from the specific moving part of the room and/or from the participant self-oscillation that still occur independently of the visual condition. Under all these visual conditions, participants stood inside the moving room at two distances from the front wall: 0.25 and 1.5 m. These distances resulted in different subtended visual angles. Considering a horizontal visual field of 200 degree [25], when participants stood at distance of 0.25 m, the horizontal visibility of each lateral wall was of approximately 23.4 degree (both sides: 46.8 degrees) and the horizontal visibility of front wall was of approximately 153.2 degrees. When participants stood at distance of 1.5 m, the horizontal visibility of each lateral wall was of approximately 65.0 degrees (both sides: 130 degrees) and the horizontal visibility of front wall was of approximately 70 degrees.

Each participant performed 13 trials, each lasting 60 seconds. In the first trial the room remained stationary. At subsequent trials, participants performed two trials at each distance, grouped under each visual condition. Therefore, each participant performed 12 trials, with flow structure and the distance order randomly assigned in each set. A resting period ranging from 30 to 120 seconds was provided after each trial in order to prevent muscle fatigue or inattentive behavior during the test. In this period participants remained seated on a chair outside the room.

Given that prior knowledge about movement of the room has been shown to have an effect on the strength of visual information and body sway coupling [26, 27], participants were not told that the room would be moving. After the test, participants were asked whether they had noticed anything unusual about the room. None of the participants noticed or were aware that the room had moved.

### Data Analysis

Some of the children were unable to accomplish the task as required by the experimenter as they moved his/her feet and/or arms during the trial producing postural sway other than the common sway as ones stands still or, in a few cases, just refused to complete the trial. In these cases, trials were repeated after all trials had been performed. Additionally, observation notes taken during data acquisition were reviewed in order to attest to the validity of each trial. A trial was considered valid and saved for future analysis when (a) the participant remained in upright position looking toward the front wall of the moving room, (b) no abrupt movements were performed, and (c) the participant performed the task for at least 30 consecutive seconds.

After completing trial screening, the following analyses were performed. Since the room oscillated in the anterior-posterior direction, analyses focused only on this direction. A frequency-response function (FRF) was employed in which constituted, first, to obtain the Fourier spectrum for each body sway and room movement. Following, the computed Fourier transforms for body sway were divided by those correspondents for room movement, resulting in a complex-valued function (transfer function) for each participant at each trial [28].

From the transfer function values, gain and sway variability (position and velocity variability) were estimated for each trial and then averaged across groups in order to verify the visual effect of stimulus on body sway at 0.2 Hz driving frequency. Gain was computed as the absolute transfer function value indicating the strength of visual stimuli and body sway coupling. Gain values of 1 indicate that the trunk sway amplitude spectrum was the same as that of the moving room, and values below and above 1 indicate that body sway had a smaller or larger amplitude spectrum, respectively, than that of the moving room.

Position and velocity variability of body sway corresponds to standard deviation and derivative of residual body sway, respectively, after removal of body sway response from sensory drive frequencies (cf. [29]). Residual body sway was calculated by subtracting sinusoids corresponding to Fourier transforms of body sway from visual stimulus frequencies.

Additionally, mean sway amplitude was obtained for all trials by estimating the mean variance of body sway. Before calculating the mean sway amplitude, a first order polynomial was subtracted from the signal at each trial. This eliminated all low-frequency changes in body sway during the trial that were not related to body oscillation. Then, the mean sway amplitude was estimated by obtaining the standard deviation for time series of body sway. Mean sway amplitude, therefore, corresponded to body sway variance and was used to determine average performance of the postural control system.

### Statistical Analysis

Because the main question of this study was to compare the use of visual cues within different conditions among children and adults, at two different distances, four multivariate analyses of variance (MANOVAs) with group (4-, 8-, 12-year-olds, and young adults) and distance (0.25 and 1.5 m) as factors, the latter treated as repeated measures, were employed in this study. Dependent measures, for each MANOVA, were mean sway amplitude, gain, position variability and velocity variability for each vision condition (global, frontal, and peripheral optical flow). Therefore, visual condition was included into the analysis but not as a factor avoiding unnecessary comparisons and interactions that would make the design more complicated. Whenever necessary, appropriate follow-up univariate analyses and Tukey post hoc tests were employed. All the analyses were performed by means of SPSS software and α-level at 0.05.

## Results

Visual information from the moving room induced body sway in all participants, under all visual and distance conditions. Results are presented below, for each variable, separately.

### Mean sway amplitude

**Fig 2:**
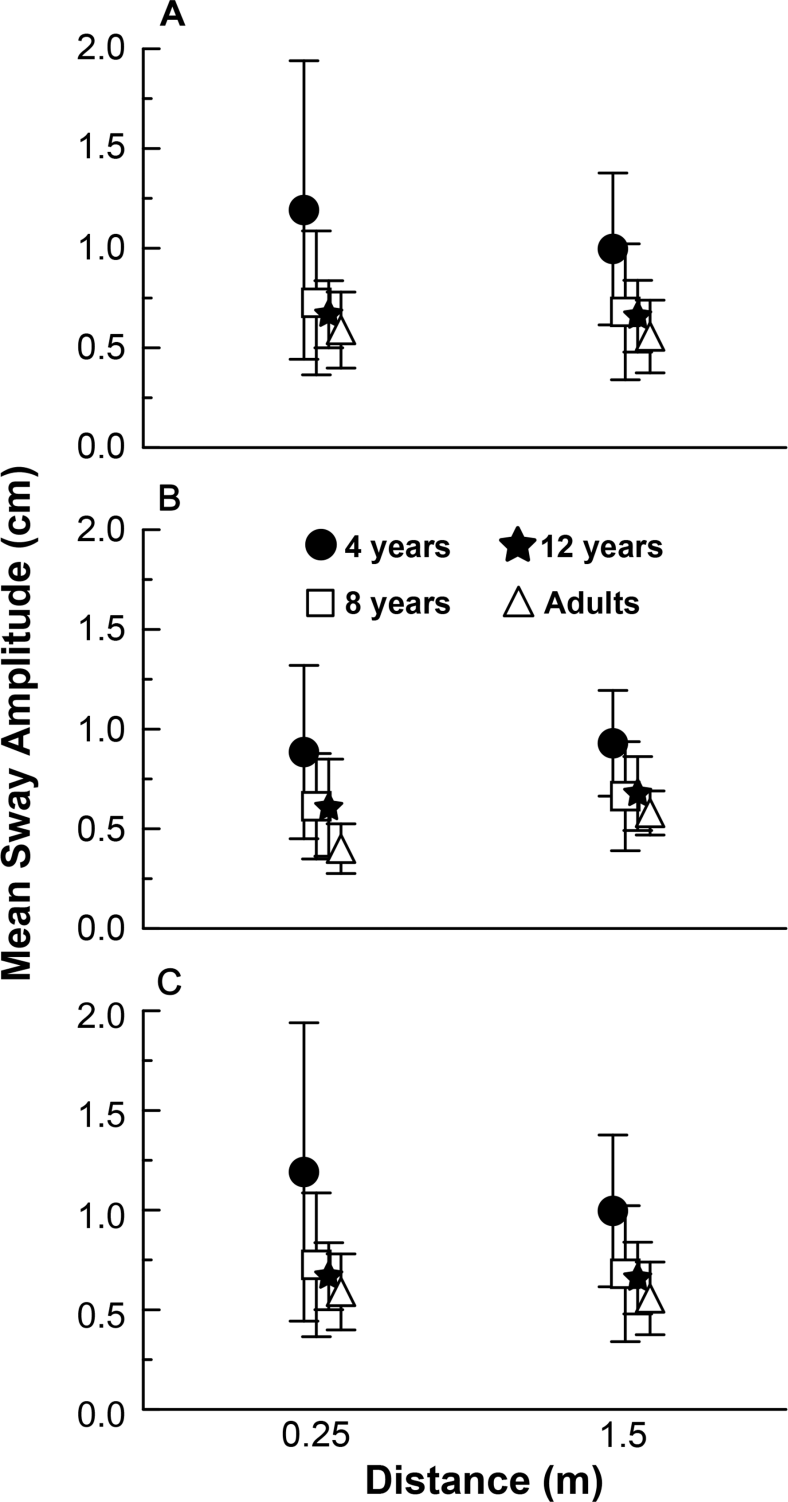
Mean sway amplitude. Mean sway amplitude values for all age groups in the anterior-posterior direction at two distances under global (A), peripheral (B), and central optical flow (C) experimental conditions.

Fig 2 depicts sway variability mean values for all age groups in the anterior-posterior direction at three distances under global optic flow (a), peripheral optic flow (b), and central optic flow (c) experimental conditions. MANOVA revealed group (Wilks’ Lambda = 0.430, F(9,82) = 2.22, p<0.05), distance (Wilks’ Lambda = 0.648, F(3,34) = 6.14, p<0.005), and group and distance interaction (Wilks’ Lambda = 0.464, F(9,82) = 3.42, p<0.005).

Univariate analyses showed that group effect occurred under global (F(3,36) = 3.00, p<0.05), peripheral (F(3,36) = 6.41, p<0.005), and central (F(3,36) = 5.43, p<0.005) optical flow conditions. Post hoc tests showed that 4-year-old children’s body sway was larger than that of adults under all optical flow conditions. Under frontal optical flow condition, 4-year-olds’body sway was also larger than that of 8- and 12-year-olds. No difference was observed for the optical flow conditions under investigationwhen8-year-olds’, 12-year-olds’, and adults’ body sways were compared. Univariate analyses showed that distance effect occurred only under peripheral condition (F(1,36) = 11.86, p<0.005), with body sway at 1.5 m larger than that at 0.25 m from the frontal wall. Finally, univariate analyses showed group and distance interaction only under global optical flow condition (F(3,36) = 11.21, p<0.001). Post hoc tests indicated that under optical flow global condition, only at 0.25 m was 4-year-old children’s sway larger than that of 8-year-olds, 12-year-olds, and adults. No difference was observed at 1.5 m from the front wall.

### Gain

**Fig 3:**
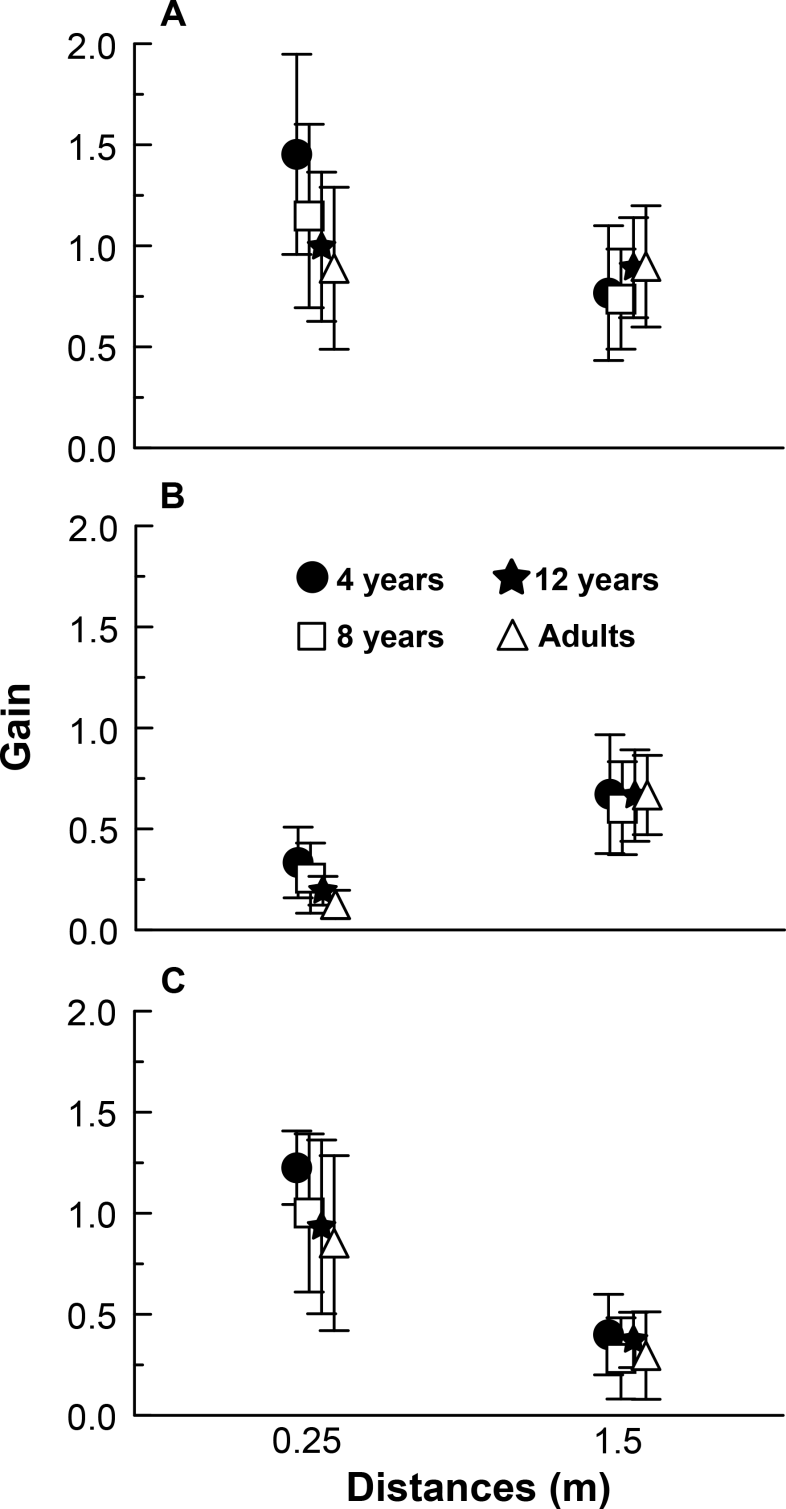
Gain between visual stimulus and body sway. Mean gain values for all age groups in the anterior-posterior direction at two distances under global (A), peripheral (B), and central optic flow (C) experimental conditions.

Fig 3 depicts gain values for all age groups in the anterior-posterior direction at three distances under global optic flow (a), peripheral optic flow (b), and central optic flow (c) experimental conditions. MANOVA revealed distance (Wilks’ Lambda = 0.063, F(3,33) = 162.82, p<0.001) and group and distance interaction(Wilks’ Lambda = 0.374, F(9,80) = 4.44, p<0.001).

Univariate analyses indicated distance effect under global (F(1,35) = 36.02, p<0.001), peripheral (F(1,35) = 229.11, p<0.001), and central (F(1,35) = 274.70, p<0.001) optical flow conditions. Under global and central optical flow conditions, induced body sway was larger at 0.25 m than at 1.5 m from the frontal wall, whereas under peripheral condition, induced body sway was larger at 1.5 m than at 0.25 m. Univariate analyses showed that group and distance interaction occurred under global (F(3,35) = 11.08, p<0.001) and peripheral optical flow conditions (F(3,35) = 5.01, p<0.01), and was marginal under central condition (F(3,35) = 2.63, p = 0.065). Post hoc tests showed that at 0.25 m from the front wall, 4-year-old children’s induced body sway was larger than that of adults under global, peripheral, and frontal conditions. Furthermore, 4-year-olds’ induced body sway was larger than that of 8- and 12-year-olds under global and frontal conditions. Lastly, 8-year-olds’ induced body sway was larger than that of adults under global optical flow condition. No difference was observed among groups at 1.5 m from the front wall.

### Position and Velocity Variability

**Fig 4:**
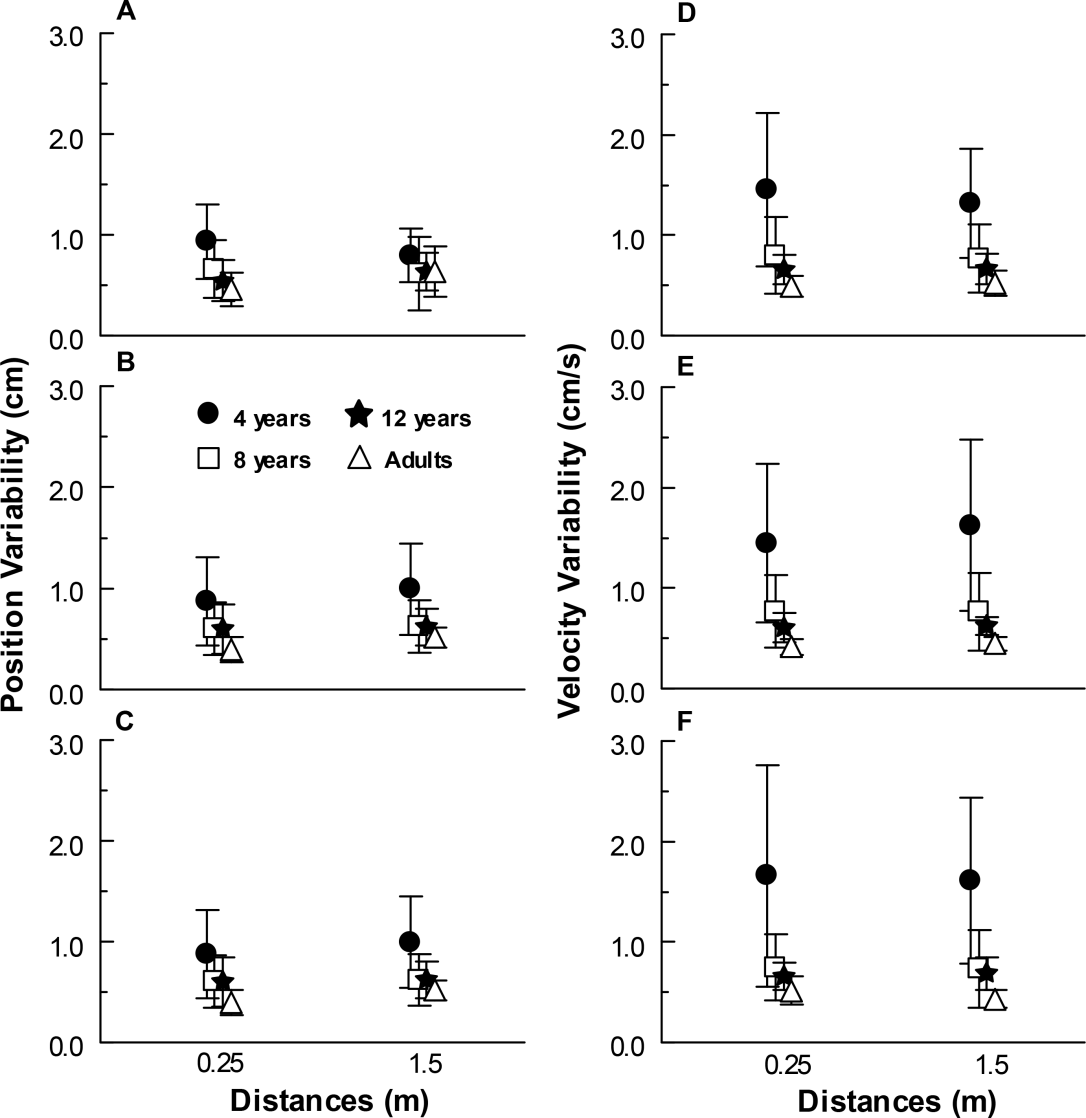
Position and velocity variability. Mean position (A-C) and velocity (D-F) variability values for all age groups in the anterior-posterior direction at two distances under global (A, D), peripheral (B, E), and central optic flow (C, F) experimental conditions.

Fig 4 depicts position (A-C) and velocity (D-F) variability values for all age groups in the anterior-posterior direction at three distances under global (A, D), peripheral (B, E), and central optic flow (C, F) experimental conditions. As regards position variability, MANOVA revealed group (Wilks’ Lambda = 0.588, F(9,82) = 2.24, p<0.05) and distance effects (Wilks’ Lambda = 0.628, F(3,34) = 6.71, p<0.005), and group and distance interaction (Wilks’ Lambda = 0.499, F(9,82) = 3.04, p<0.005). Univariate analyses showed that group influence occurred under global (F(1,36) = 3.95, p<0.05), peripheral (F(1,36) = 6.37, p<0.005), and central (F(1,36) = 5.56, p<0.005) optical flow conditions. Post hoc tests indicated that position variability under global and peripheral optical flow conditions was higher for 4-year-oldsas compared to adults and that under frontal condition, 4-year-olds’ position variability was higher than that of older children and adults. Univariate analyses showed that distance effect occurred under global (F(1,36) = 8.67, p<0.01, peripheral, F(1,36) = 7.02, p<0.05) and central (F(1,36) = 11.20, p<0.005) optical flow conditions. Under both peripheral and frontal optical flow conditions, position variability was lower at 0.25 m than at 1.5 m, whereas under global optical flow condition, position variability was higher at 0.25 m than at 1.5 m from the front wall. Univariate analyses showed that only under global (F(3,36) = 10.45, p<0.001) condition did group and distance interaction occur. Post hoc tests indicated that at 0.25 m from the front wall, 4-year-olds’ position variability was higher than that of adults. No difference was observed in other group comparisons at 0.25 m from the front wall. Finally, no differences were observed among groups at 1.5 m from the front wall.

As regards velocity variability, MANOVA revealed group effect (Wilks’ Lambda = 0.305, F(9,82) = 5.79, p<0.001) and group and distance interaction (Wilks’ Lambda = 0.581, F(9,82) = 2.30, p<0.05). Univariate analyses showed that group effect occurred under global (F(3,36) = 15.58, p<0.001), peripheral (F(3,36) = 22.55, p<0.001), and central (F(3,36) = 19.37, p<0.001) optical flow conditions. Post hoc tests indicated that speed variability was higher for 4-year-oldsas compared to older children and adults under all three optical flow conditions and higher for 8-year-oldsas compared to adults under peripheral optical flow condition. No differences were observed in other group comparisons. Univariate analyses showed that group and distance interaction occurred only for frontal optical flow (F(3,36) = 7.68, p<0.001). Post hoc tests indicated that, at both 0.25 and 1.5 m from the front wall, velocity variability was higher for 4-year-old children as compared to adults. No difference was observed in other group comparisons under both distance conditions.

## Discussion

This study investigated the effect of distance and optical flow structure on visual information and body sway coupling in children and young adults. The results indicate that the effect of different distances between participants and the front wall of the moving room on their use of visual information to control body sway is age dependent. Optical flow structure provides different visual information, leading to age-dependent visual information and body sway coupling of varying strength. These results suggest that the way in which children integrate visual cues with sensory stimuli coming from other channels to control body sway changes throughout the first decade of life.

### Effect of distance and optical flow structure on corresponding body sway

The results from this study clearly show that visual cues are used differently by younger children as compared to older children and adults to control body sway at different distances from the front wall of the moving room. At a shorter distance from the front wall (0.25 m) and under global optical flow condition, 4-year-old children were the ones influenced the most by visual stimulus manipulation (highest gain values) as compared to 8- and 12-year-olds and young adults. Following the same trend, 8-year-old children were more influenced than adults were. Overall, these results indicate that younger children, when exposed to a condition in which visual cues are stronger, such as global flow structure and at 0.25 m from the front wall, use visual stimulus by assigning a higher weight to it, which increases its effect on body sway. Interestingly, at a farther distance and under global flow condition, no such difference was observed among children and adults. The effect of distance of visual cues on body sway has been previously observed in children as compared to adults under global flow condition [16] as well as under monocular and binocular conditions [30].

Besides corroborating previous results, the results from this study also show that age-distance dependence is unrelated to optical flow structure. Although different effects of flow structure on body sway have not been directly compared, it is possible to verify that gain values (Fig 3) vary with differing optical flow conditions, as previously observed [19, 20]. Akin to older children and adults, 4-year-olds were influenced by flow structure. Moreover, while differences in using visual optical flow cues have been observed early in life [9, 17, 18], they were not observed at the age of 4 years.

Results from this study suggest that despite 4-year-olds being influenced by different optical flow visual cues in the same way as older children and adults are, the former respond differently from the latter under stronger stimuli under peripheral and central flow conditions. Interestingly, none of these age differences were observed under optical flow condition at 1.5 m from the front wall. Therefore, whereas young children have developed the capability of using visual cues under different optical flow conditions, they are not yet capable of using visual cues of differing strengths. It is worth mentioning that, at a shorter distance from the front wall, 4-year-olds respond more strongly than 8- and 12-year-olds do under global and central optical flow conditions, but not under peripheral condition, in which just 4-year-old children were more influenced than adults. Considering a horizontal visual field of 200 degree [25], at distance of 0.25 m, the horizontal visibility of each lateral wall was of approximately 23.4 degree (both sides: 46.8 degrees), and at distance of 1.5 m, the horizontal visibility of each lateral wall was of approximately 65.0 degrees (both sides: 130 degrees). Thus, the peripheral optical flow condition, as expected, was the least effectual condition, with lowest gain values, indicating that visual stimuli were not as strong as they were under the other two conditions.

Several studies have indicated that sensorimotor integration changes during the course of the first years of age and young children are unable to properly integrate sensory cues deriving from multiple sensory systems [2, 16, 31, 32] and only reach adult-like integration after the first decade of life [32–34]. These studies have manipulated the availability or quality of sensory cues provided to children. Based on the results presented in this study, it should be added that the difficulty in accommodating sensory cues changes are less likely related to optical flow and most likely related to the strength with which visual cues carry information in children.

### Coupling/uncoupling to visual information

Besides being more influenced by visual stimulus at a short distance, 4-year-old children’s body sway was larger (Fig 2) under all optical flow conditions and at both distances (0.25 and 1.5 m) from the front wall as compared to adults as well as 8- and 12-year-olds in many cases. Moreover, this larger sway magnitude occurred at frequencies other than that of the driving signal, leading to higher position and velocity variability (Fig 4). In both measures, a quite variable behavior was observed among the 4-year-old children (Figs 2 and 4 large SD for 4-year-old children), indicating that postural sway and the variability inherent to this sway varies dramatically from one to other. Such a high variable behavior might be a signature of developmental changes that occurring at a relatively short period of time, might lead to different behavior even among children with close chronological age.

Barela, Clark, and Jeka [8] have suggested that young children have difficulty in uncoupling to irrelevant sensory stimuli and, as a result, are unable to focus on a specific and more useful sensory stimulus to perform a task. This results in a postural performance that inherently shows higher variability. Young children’s larger sway magnitude when standing upright has been a consistent and common finding [1, 2, 6, 35] and the results presented in this study corroborate prior observations. They also corroborate previous results [8] indicating that 4-year-olds’ sway is characterized by higher variability; as observed in the present study regarding both position and velocity components under all three visual optical flow conditions and at 0.25 and 1.5 m distances from the front wall.

Therefore, irrespective of the visual stimulus properties, younger children swayed to components other than those conveyed by the driving sensory manipulation. The higher level of inherent noise during response to driving signal may be attributed to young children’s immature process of reweighting sensory information from different sources in order to internally assess body orientation [8]. Indeed, it has been demonstrated that young children, including 4-year-olds, do not properly calibrate reweighting responses when sensory stimulus properties change [12, 32]. Nevertheless, the results from this study further advance knowledge on the reweighting process and response noise level in view of the fact that variability in body sway was about the same at both 0.25 and 1.5 m from the front wall and under all visual optical flow conditions (Fig 4). Conversely, differences in body sway responses (Fig 3) were observed for 4-year-old children at different distances under all optical flow conditions. For instance, although young children are able to reweight their body sway response based on stimulus condition, there is no difference in sway variability. Moreover, in doing so, younger children sway with larger magnitude (Fig 2).

Swaying in response to the moving room induces conflict with other sensory sources, given that somatosensory and vestibular cues provide different information from that delivered by visual stimuli. Recent evidence has demonstrated that not only do adults change their responses to changing stimuli (intra-modality reweighting), but they also concomitantly change their responses to other stimuli that have not changed (inter-modality reweighting) [36]. Despite the fact that no previous studies have confirmed this reweighting process in children, looking at inter-modality reweighting in children, the results presented in this article shed light on this issue. Even 4-year-old children were capable of changing their response to accommodate varying optical flow structures and distances from the visual stimulus, with gain values changing accordingly. Despite this sensory reweighting in response to visual stimuli, both position and velocity variability remained unchanged. Thus, while younger children down/up-weighted visual influence, body sway was still embedded with characteristics other than those conveyed by visual stimuli. This being the case, even though younger children can produce intra-modality sensory reweighting, as previously demonstrated [12, 32], they may not be able to change along with other sensory modality influences as adults do [36], leading to sway characterized by higher variability.

Developing flexible and accurate coupling between sensory stimuli and motor responses is not trivial and requires that the central nervous system determine which sensory information is relevant to the task. The literature presents many instances of infants’ and young children’s difficulties in managing sensory conflicting situations. A classic example comes from pioneering studies with moving rooms in which infants’ upright position is radically disrupted upon exposure to visual stimulus manipulation[14]. Likewise, young children are not able to maintain upright orientation under complex sensory conflicting conditions [37]. Therefore, it seems that children’s central nervous system calls for repetitive exposure to conflicting conditions over the first years of life for them to learn to ignore inaccurate stimuli and focus solely on those that are relevant and provide reliable information to properly perform the task. Thus, embedded in the process of sensory integration from multiple sources is a reweighting process that involves reducing/increasing the effect of specific sensory information (intra-modality) as well as increasing/reducing the effect of other sensory information (inter-modality).

Our results suggest that children as young as 4 years old are capable of tackling with different optical conditions. Notwithstanding, they still exhibit differences in response to stimuli characterized by varying information strengths. Moreover, even in situations in which younger children are able to compensate for visual changes by responding in the same way as older children and adults do, their behavior varies more. This study suggests that higher variability in young children’s body sway might be due to their difficulty in reweighting all sensory influences at the same time, given the availability of diverse sources of stimulus (inter-modality reweighting). We suggest that these issues need to be further examined in experimental settings with direct manipulation of these aspects and with more and longer trials performed by children. Such suggestions are not trivial but would provide a better understanding of the development adaptive process imbedded in how children developmentally shape the use of available sensory cues to control posture.
